# The Landscape of Sexual Harm in the Video Game, Streaming, and Esports Community

**DOI:** 10.1177/08862605241271349

**Published:** 2024-08-21

**Authors:** Oliver J. Merry, Kate C. Whitfield

**Affiliations:** 1 Sheffield Hallam University, South Yorkshire, UK

**Keywords:** sexual assault, sexual abuse, child abuse, internet and abuse, sexual harassment

## Abstract

While sexual harm has been studied across a range of contexts, it has not yet been considered within the video game, streaming, and esports community. This study aimed to explore the landscape of sexual harm in this community, specifically, where it has been committed by esports professionals and video game live streamers. Fifty-five victim statements were extracted from online sources (such as Twitter/X and Reddit) and coded into variables relating to offender and victim demographics, offense characteristics, the offense process, and platform(s) used. Descriptive statistics were generated for each variable and Fisher’s exact tests were conducted to examine the differences between adult-on-adult and adult-on-child cases. The findings reveal diverse offense outcomes across the sample, ranging from rape to sexual communication with a child. Some offense patterns can be seen in wider sexual offending literature, such as pre-offense alcohol consumption, offending against incapacitated victims (e.g., sleeping), and offending within an established romantic relationship. However, several offense process characteristics unique to the video gaming community were identified. These included offenders using their position of fame within the community to access victims and bypass the need for other coercive behaviors. Online offenses were more common with children and offenders demonstrated a preference for “live” methods, such as voice chat and video calling, rather than instant messaging or sharing images of themselves. This limits the digital evidence left behind and indicates the offenders’ greater technological literacy. The study’s findings shed light on the sexual harm that exists within this previously unexplored context and highlight areas where esports organizations, live-streaming platforms, and educational providers can do more to safeguard players, fans, and viewers in this community.

The negative consequences of sexual offense victimization are significant, including depression, suicide, anxiety, post-traumatic stress disorder, loss of financial income, and future sexual offending (Dworkin et al., 2017, 2023; Lindert et al., 2014; Loya, 2015; Whitaker et al., 2008). As such, these offenses and behaviors have been studied across a wide range of contexts and populations, including adult-on-adult (Abbey & Jacques-Tiura, 2011), adult-on-child (Wolak & Finkelhor, 2013), stranger-on-stranger (Ellis et al., 2017), online offenses (Kloess et al., 2019), female offenses (Almond et al., 2017), and offenses within workplaces (Stone et al., 2019). However, one context that has not been explored is sexual harm through video games, streaming, and esports. Such research is important, as it is a context heavily populated by young and vulnerable people, with platforms giving potential offenders easy access to them. Consequently, these individuals may be at risk from both others within the community, as well as external parties that use video game-related spaces to identify and approach potential victims. The aim of this study is to explore the landscape of sexual harm in this community, including the potential use of unique offense patterns and behaviors. This is the first research of its kind and is therefore significant in raising awareness of the risks of sexual harm and identifying actions to help protect members of the video game, streaming, and esports community.

## Technology-Assisted Sexual Violence

With the increasing development of new technologies, the landscape of sexual offending has also evolved (Fisico & Harkins, 2021). Technology has removed the requirement for physical closeness between offender and victim for sexual harm to be caused. In relation to child sex offenses (CSO), online image-based offenses against children have existed for decades in the form of making, distributing, and/or possessing child sexual abuse material (CSAM; Crown Prosecution Service, 2020). These offenses are facilitated by technology through image-sharing and video-streaming websites and file-sharing technologies (e.g., e-mail and peer-to-peer sharing). Technology has made CSAM more accessible, with research indicating a 50% annual increase in CSAM offenses over the past 20 years (Bursztein et al., 2019). This increased accessibility has also been linked to an increase in abuse severity depicted (Salter & Whitten, 2022).

It is not only image offenses that are linked to new technologies. Technology is frequently used in the commission of other child sex abuse offenses, such as grooming (O’Connell, 2003). It is well documented that offenders make use of online chatrooms (O’Connell, 2003; Williams et al., 2013) and social media sites (Weingraber et al., 2020) to sexually groom children. The outcome offense of grooming can vary, from exposure to pornographic material, to self-produced indecent images, cybersex, and meeting in person for a contact sexual offense (DeHart et al., 2017).

Children are not the only ones at risk of technology-assisted sexual violence. Adults can also be the victim of online image-based offenses. For example, revenge porn refers to the “non-consensual distribution of private, sexual images by a malicious ex-partner” (McGlynn et al., 2017, p. 26), which has been shown to cause feelings of shame, humiliation, depression, and anxiety in victims (Bates, 2017). Further, the offense does not need to take place online for it to be technology-assisted. Research has demonstrated offenders using technology, such as dating apps, to meet victims and lure them to an in-person meeting where a contact offense takes place (Pooley & Boxall, 2020). In an Australian survey, one-third of respondents (49.5% of women) disclosed experiencing sexual violence that began on a dating app (Wolbers et al., 2022).

More recently, developments in virtual reality (VR) provide offenders with a pseudo-physical virtual environment where physical contact can be mimicked, further enhancing the possibility for sexual harm to take place online. For example, within an hour of exploring the virtual environment of “Horizon Worlds,” a researcher was led to a private room at a virtual party, where an avatar simulated raping her avatar while others watched (Ekō, 2022). While research regarding VR as a tool for sex offending is limited, charity organizations warn about the scope of sexual harm through this new technology. This includes simulating child sexual abuse and creating synthetic CSAM (WeProtect Global Alliance, 2023).

Technological advancements have broadened the landscape of sexual harm. In the United Kingdom (UK), the government has developed the Online Safety Bill, which aims to protect children and adults online by legislating against technology-assisted sexual violence offenses. This includes forcing social media companies to be more proactive in protecting their users and seeks to criminalize new harmful behaviors (Department for Science, Innovation and Technology & Department for Digital, Culture, Media and Sport, 2023). However, most of the focus of the Bill centers around social media companies. While this is important, as social media is a prevalent tool used by offenders to cause sexual harm (Choo, 2009), there is little consideration of the use of video games and other gaming-related platforms.

## Video Game Live Streaming and Esports

Video games have developed considerably over the past few decades and have attracted growing interest from the population. The majority of children (93%) are engaged in some form of video gaming (Children’s Commissioner, 2019), while 50% of adult men and 48% of adult women play some form of video game (Duggan, 2015). Due to the popularity of video games, the field is constantly developing. Cabeza-Ramírez et al. (2021) argue that the world of video games is currently experiencing a revolution. This revolution is predominantly driven by two recent, yet interconnected genres of content; video game live streaming and esports (Cabeza-Ramírez et al., 2021). Online sites, such as Twitch, provide platforms for anyone to live stream themselves doing their hobbies, including playing video games (Cabeza-Ramírez et al., 2022). Twitch currently holds over 240 million monthly active users, with close to 100,000 ongoing streams at any given time (Dean, 2024). Sites like Twitch then generate revenue by broadcasting advertisements on the channels of users currently streaming (Cabeza-Ramírez et al., 2022). In turn, the streamers themselves can monetize their own content through fan subscriptions (audience members paying a monthly fee to support their favorite streamer), donations (one-off payments from audience members to the streamer), and sponsorships from companies wanting to use the streamer’s platform to advertise their own product (Cabeza-Ramírez et al., 2022). Unlike traditional media, such as television and cinema, streaming platforms provide chat functions so that the audience can directly communicate with the streamer they are watching. As such, this form of entertainment can also be regarded as a social experience, which has led to the rise of distinct communities and subcultures (El Afi & Small, 2021).

Similarly, the rise of esports has also capitalized on the exponential growth of video games. Esports represents organized competitive video gaming (Rogstad, 2021), often involving regular teams and players who attract their own fanbase and takes place in front of an audience (either in person or live streamed). There is significant variation in definitions of esports; however, it is generally agreed that esports refers to “competitive multiplayer gaming that involves spectating real-time or asynchronous gameplay, team competitions, and tournaments either online or offline” (Wohn & Freeman, 2020, p. 73). As with traditional sports, esports offers a diverse range of levels (e.g., amateur, collegiate, professional) and genres (e.g., first-person shooter, real-time strategy, and racing). Both live streaming and esports offer players a way to “professionalize” their hobby, making playing video games their source of income.

Live streaming and esports have coevolved together, with researchers arguing the success of esports has been facilitated by the emergence of live streaming (Wohn & Freeman, 2020). Consequently, Wohn and Freeman (2020) state that studying video gaming, live streaming, or esports independently of one another fails to provide a comprehensive understanding of how people actually engage with this form of media. Instead, playing, viewing, spending, and live-streaming video games should be considered a multidimensional media ecosystem.

## Sexual Harm in Video Games

Despite the growing engagement and investment in video games, live streaming, and esports, there is a considerable lack of research regarding the sexual harm that occurs in video games and on gaming-related platforms. The studies that do exist tend to focus on sexual harassment and sexist comments, made by male players toward female players (Cote, 2017; Fox & Tang, 2014, 2017; Oh & Choi, 2023; Ruvalcaba et al., 2018; Seo et al., 2022). This research demonstrates that despite a growing female player base, video games are still stereotypically viewed as straight, white, male spaces (Cote, 2017; Fox & Tang, 2014). Players seen as “outsiders” are then targeted for harassment. Several factors feed into this. Fox and Tang (2017) propose the online disinhibition effect (Suler, 2004), where the anonymity, lack of verbal cues, and lack of legitimate authority in video game environments lessen the restraints of social norms, making players feel more comfortable to act in an abusive manner. Additionally, Cote (2017) argues the culture in competitive video games plays a contributing role, as players view “trash talking” as a distinct, and even inevitable, part of the game. This has led to a culture where harassment is ignored, excused, or not taken seriously, as it is perceived as being part of the gaming experience (Cote, 2017).

The limited research available on the experiences of live streamers shows gendered differences in the content of viewers’ comments in streamers’ live chat. Ruvalcaba et al. (2018) found female streamers were over 10 times more likely to receive directed sexual comments. Additionally, while women receive more positive comments than men, a significant portion of these comments are about their physical appearance.

The impact of harassment is significant on the victimized player base. Cote (2017) identified that women playing online video games employ a range of strategies to reduce their risk of harassment, including avoiding playing with strangers, hiding their gender in games, and quitting online gaming altogether. When harassed, different coping strategies are used, including blocking the offending player, retaliation, and relying on gaming skills to silence criticism.

Beyond these research findings, “VTubing” is becoming increasingly popular. This involves streaming while using an interactive 2D or 3D animated avatar that mimics streamers’ real expressions and reactions. This allows the streamer to hide their real physical appearance, with anecdotal evidence suggesting it adds an extra layer of protection between the streamer and their audience, relieving some of the pressure on female streamers to meet sexist expectations regarding physical appearance (Williams, 2022).

The limited research regarding online gaming and streaming paints a picture of a sexist virtual environment that is hostile toward women. This is concerning, as there is a correlative link between hostile sexism and sexual aggression (Bosson et al., 2015). However, to date, there is no research that considers sexual harm committed in video game communities, including live streaming and esports. As such, the aim of this study is to explore the landscape of sexual harm in the video game, streaming, and esports community. Specifically, this research seeks to explore the range of sexual harm victimization in this community, as well as understand the offense processes employed by offenders in this context. Such findings would allow comparisons to be drawn with the wider literature, as well as the potential identification of unique offense patterns employed in the video game, live streaming, and esports community. For example, news media have reported cases of live streamers using their position of influence and fame in the community to victimize their fans (Sacco, 2021), and esports coaches using their position of power over players to sexually offend (Nightingale, 2022). This is important to advance understanding regarding the sexual harm reported within this community, raise awareness of the risks of sexual harm, and identify protection measures to help prevent harm.

## Methods

### Sample

The sample consisted of 55 victim statements, totaling over 70,000 words (*M* = 1,292). The statements described 55 sexual offenses committed by 45 offenders. Of the offenders, the majority were described by victims as male (*n* = 41, 91.1%), with three (6.7%) female and one (2.2%) transgender. Eight (17.8%) offenders were described by victims as LGBTQ+. At the time the offense began, 44 (97.8%) offenders were aged 18 or above, and one (2.2%) offender was under the age of 18. The precise age of offenders at the time of the offense was available in 20 cases and ranged between 17 and 41 years (*M* = 25.2, *SD* = 5.66). The majority of offenders were described as living in the United States of America (USA; *n* = 35, 77.8%), with six in the UK (13.3%), and one (2.2%) each in Brazil, The Netherlands, Poland, and Serbia. In terms of involvement in the video game community, 34 (75.6%) offenders engaged in esports (e.g., as a player, coach, or manager), and 18 (40%) streamed video game content (e.g., on platforms such as Twitch, YouTube, or Facebook). Eight (17.8%) offenders took part in both esports and streaming. Additionally, one offender was neither involved in esports nor streaming but instead managed prospective streamers.

In terms of the victims, the majority identified as female (*n* = 44, 80%), with seven (12.7%) male, three (5.5%) non-binary, and one (1.8%) transgender. Nine (16.4%) of the victims identified as LGBTQ+. At the time the offense began, 33 (60%) victims were aged 18 years or above, and 22 (40%) were under the age of 18. The precise age of the victims at the time of the offense was available in 30 cases and ranged between 13 and 30 years (*M* = 16.7, *SD* = 3.4). Location data for victims was available in 54 cases and is similar to that of the offenders, with the majority of victims living in the USA (*n* = 38, 70.4%) and UK (*n* = 9, 16.7%), and 1 (1.9%) each in Argentina, Brazil, Canada, the Netherlands, Poland, Serbia, and Sweden. In terms of involvement in the video game community, 20 (36.4%) victims streamed video game content and 17 (30.9%) engaged in esports.

Across the 55 offenses, 22 (40%) were sex offenses against a child, which involved a victim under the age of 18 years and an offender aged 18 or above. The remaining 33 (60%) cases involved adult victims and offenders.

### Data Collection

Following the receipt of ethics approval from the institution’s research ethics committee (ER47925052), data were collected from social media and related platforms. Over the past 5 years, the video gaming industry and community has experienced its own #MeToo moment. In the summer of 2020, more than 70 allegations surfaced on social media detailing a range of sexual victimization (Lorenz & Browning, 2020). Since then, more people have come forward to share their experiences of sexual victimization online. These allegations often take the form of victim statements written on sites such as Twitlonger (a site that allows users to write long-form content to share on Twitter, now known as X) or GoogleDocs, which are then shared publicly on social media sites, such as Twitter/X and Reddit. The detail and length of the statements vary; however, a significant proportion of them include some form of evidence, such as screenshots of conversations. These statements represent a rich data source on a highly sensitive topic, providing significant detail on the offense(s) and surrounding circumstances. The statements were extracted for the current study and no contact was made with any of the individuals involved.

Unobtrusive research methods such as this have several drawbacks, including selective recording, being from a single perspective, inability to request further detail, and not being written for research purposes (Alison et al., 2001; Kellehear, 1993). However, despite these drawbacks, unobtrusive methods offer access to rich data on sensitive topics, where the level of participants’ frankness is unlikely to be achieved through conventional qualitative methods (Hine, 2011). Additionally, as is the case in the present study, unobtrusive methods offer insights into areas with limited access and where no previous research exists.

Statements were identified through Google by using the following search terms: “esports sexual offence,” “esports sexual assault,” “video game streamer sexual assault,” and “video game streamer sexual offence.” These searches led to items on video gaming-specific news outlets, which provided a direct link to the statement. Similarly, several Reddit threads were also identified, where users had compiled a list of links to Twitter/X posts of victims sharing their accounts. The statements and their relevant HTML links were copied and stored in a data corpus, where they were subsequently anonymized. Only long-form statements were included in the study (such as those on TwitLonger or in Google Docs). Some statements were single Twitter/X posts, and these were excluded due to the character limitations not allowing sufficient detail.

By consulting the existing literature on sexual offending (e.g., Almond et al., 2020; Bergen et al., 2014; Canter et al., 2003; Priebe & Svedin, 2009; Schulz et al., 2016) and reading through the statements, a number of variables were identified that relate to offender and victim demographics, offense characteristics, the offense process, and platform(s) used. All identified variables were incorporated into a coding dictionary, which defined the variables and continued to be developed iteratively. For example, some codes were split into additional codes to preserve a greater level of detail. This led to the identification of 94 variables, which were subsequently coded in each of the 55 cases. Coding was primarily dichotomous, where present variables were assigned a code of 1 and absent variables were assigned a code of 0. Several multinomial variables were also coded (e.g., offense location, platforms used), as well as some continuous variables (e.g., age and duration of offense). No identifiable information was collected through the coding process to ensure victims maintained anonymity. Additionally, no direct quotes from the statements were used in the reporting of results so they cannot be used to trace the original statements and potentially identify the victims.

### Data Analysis

As no previous research exists in this area, data analysis needed to be exploratory. Once the data had been coded, descriptive statistics were generated for each variable in the coding dictionary. Fisher’s exact tests were then conducted to examine the differences between adult-on-adult and adult-on-child cases. A *p*-value smaller than .05 was considered statistically significant.

## Results

### Offending Behavior

Both in-person and online offending behavior were recorded. In-person offending behavior is related to unwanted and non-consensual physical acts directed toward the victim by the offender. In terms of online behavior, some of the actions do not constitute an offense unless committed against a young person. However, where the incident involved two adults, the online behavior was part of the process that led to the in-person offense. The frequency of offending behavior across the cases is presented in Table 1. The findings are split into adult sex offenses (ASO) and CSO to allow comparison. Significant differences between ASO and CSO cases are also indicated in the table.

**Table 1.** Frequency of In-Person and Online Offending Behavior.

Offending behavior	ASO (*n* = 34)		CSO (*n* = 21)		Total (*N* = 55)	
	*f*	%	*f*	%	*f*	%
In-person						
Non-sexual touching***	18	54.5	2	9.1	20	36.4
Covered touching	13	39.4	4	18.2	17	30.9
Uncovered touching	12	36.4	3	13.6	15	27.3
Rape*	12	36.4	2	9.1	14	25.5
Forced kissing	11	33.3	2	9.1	13	23.6
Forced undressing*	11	33.3	1	4.5	12	21.8
Sexual harassment*	8	24.2	0	0.0	8	14.5
Digital penetration	7	21.2	1	4.5	8	14.5
Indecent exposure	5	15.2	1	4.5	6	10.9
Attempted rape	2	6.1	0	0.0	2	3.6
Online						
Sent sexual communication (IM)***	1	3.0	19	86.4	20	36.4
Request sexual communication (IM)***	0	0.0	16	72.7	16	29.1
Request sexual media of victim***	1	3.0	12	54.5	13	24.1
Sent sexual communication (VoIP)***	0	0.0	12	54.5	12	21.8
Cybersex masturbation***	1	3.0	10	45.5	11	20.0
Request sexual communication (VoIP)***	0	0.0	9	40.9	9	16.4
Request cybersex masturbation***	0	0.0	8	36.4	8	14.5
Sent sexual media of self**	0	0.0	5	22.7	5	9.1

*Note*. ASO = adult sex offenses; CSO = child sex offenses; IM = instant messaging; VoIP = Voice over Internet Protocol.

* *p* ≤ .05. **p ≤ .01. ***p ≤ .001.

With regard to in-person behavior, rape was detailed in just over a quarter (25.5%) of cases. Additionally, Fisher’s exact test results show a significant difference between adult and child cases in terms of non-sexual touching (*p* = .001), rape (*p* = .029), forced undressing (*p* = .018), and sexual harassment (*p* = .016), with adult victims experiencing these behaviors more than young people.

However, in terms of online behavior, there is a noticeable change in Fisher’s exact test results, with significant differences between adult and child cases being found with regard to all online behaviors, with young people experiencing them a lot more frequently than adults. The offender sending sexual communication through instant messaging (IM), often referred to as “sexting,” was particularly common in cases involving a child (86.4%). Similarly, requesting sexual communication through IM from the victim was also very frequent (72.7%). Sexual communication also occurred via Voice over Internet Protocol (VoIP) technology (e.g., on platforms such as Skype and Discord), often referred to as “voice chat.” The offender sending sexual communication through VoIP was outlined in 54.5% of cases involving a child while requesting sexual communication through VoIP from the child was also frequent (40.9%). Similar frequencies were also identified for cybersex (masturbating while communicating with the victim), with it being outlined in 45.5% of cases involving a child, and in 36.4% of cases, the offender asked the child to reciprocate. In terms of image-based offenses, 22.7% of offenders sent sexual images of themselves to a young person; however, a higher frequency of requests for sexual images was found, with offenders asking young people to send sexual images of themselves in 54.5% of cases.

### Offense Process Characteristics

Offense process characteristics are features of the offense that aided the offender in committing the offense. The frequency of offense process characteristics across the cases is presented in Table 2, as well as significant differences between ASO and CSO cases. The most common characteristic was persistent attempts to proposition the victim (65.5%). This created an environment for the victim where they felt unable to say “no” to the offender, as each time they said “no” it was ignored. The second most common characteristic was the offender making use of their fame status (49.1%), as many of the offenders are prominent members of their gaming communities, with a significant number of fans and admirers.

**Table 2.** Frequency of Offense Process Characteristics.

Offense process characteristics	ASO (*n* = 34)		CSO (*n* = 21)		Total (*N* = 55)	
	*f*	%	*f*	%	*f*	%
Persistent attempts	23	69.7	13	59.1	36	65.5
Fame status	15	45.5	12	54.5	27	49.1
Offender consumed alcohol**	18	54.5	3	13.6	21	38.2
Victim consumed alcohol***	16	48.5	1	4.5	17	30.9
Gifts	7	21.2	10	45.5	17	30.9
Position of power	8	24.2	5	22.7	13	23.6
Demeaning comments	4	12.1	9	40.9	13	23.6
Victim asleep*	11	33.3	1	4.5	12	21.8
Secrecy	5	15.2	7	31.8	12	21.8
Romantic relationship	5	15.2	5	22.7	10	18.2
Negative inducements	4	12.1	6	27.3	10	18.2
Suicide threat	3	9.1	5	22.7	8	14.5
Deleted evidence**	0	0.0	6	27.3	6	10.9
Insults	3	9.1	3	13.6	6	10.9
Blackmail	2	6.1	3	13.6	5	9.1
Victim consumed drugs	2	6.1	0	0.0	2	3.6
Offender consumed drugs	2	6.1	0	0.0	2	3.6

*Note*. ASO = adult sex offenses; CSO = child sex offenses.

* *p* ≤ .05. **p ≤ .01. ***p ≤ .001.

The consumption of alcohol by the offender (38.2%) and victim (30.9%) was also found in a sizable proportion of statements. Both were more likely to occur in adult cases (offender = 54.5%, victim = 48.5%) than cases involving children (offender = 13.6%, victim = 4.5%). The consumption of drugs was far less common (6.1% for both offenders and victims) and only occurred in adult cases. The alcohol and drug variables were predominantly observed as part of the in-person offenses, with the offender often buying or supplying alcohol or drugs for the victim. Additionally, in 21.8% of cases, the offender waited for the victim to fall asleep before committing the offense. This was significantly more common in adult cases (33.3%) than cases involving children (4.5%). Each of these variables demonstrates efforts by the offender to reduce the likelihood and capability of the victim to physically resist their advances.

A range of variables relating to the intrinsic power dynamic between offender and victim were identified. Almost half (49.1%) of offenders made a point to highlight their status of fame (as a live streamer or esports professional) to their victim (either subtly or overtly). This seemed to be used to make the victim feel grateful or lucky to have contact with the offender. Many victims were also worried about potential backlash from fans should they make any disclosures about the offender’s behavior. In 23.6% of cases, the offender was described as being in a position of power (e.g., a coach, manager, or boss). Within these relationship dynamics, various power-related behaviors regarding reward and punishment were also identified. Gift giving was noted in 30.9% of cases, while blackmail (the expressed threat of punishment should the victim not comply, such as sharing images) and negative inducements (the unexpressed threat of punishment) were described in 9.1% and 18.2% of cases, respectively. These power-related behaviors tended to be more common in cases involving children, although the difference between ASO and CSO cases was not significant. The gifts offered to victims varied, ranging from cash-giving, to the offer of coaching in a specific video game, the promise of helping the victim gain sponsorship deals, and offering a moderator role in their Twitch or Discord communities.

Tactics to coerce and maintain control over the victim were also recorded, such as pressuring the victim to keep their interactions secret (21.8%) and deleting evidence (10.9%), such as chat logs. Fisher’s exact test results show a significant difference between adult and child cases in terms of deleting digital evidence (*p* = .003), with it only occurring in cases that involved a child. Tactics to increase compliance included using demeaning language (23.6%) and insults (10.9%), often to lower the confidence of the victim. Additionally, making suicide threats (14.5%) if the victim resisted offender requests were also identified. All of these tactics were more common in cases involving children, although the difference between ASO and CSO cases was not significant.

### In-Person Offense Location

In total, 37 (67.3%) statements outlined an in-person offense. Table 3 presents the frequency of these offense locations across the cases. Where offenses took place in person, the most common offense locations were gaming tournaments or conventions (27.3%). This also included after-parties and/or hotels where the offender or victim was staying while attending the tournament or convention. Just under a quarter (23.6%) of the statements outlined an offense that occurred at the offender’s home residence, which was significantly more common (*p* = .008) in adult cases (36.4%) than in cases involving a child (4.5%). A further 18.2% of statements described offenses taking place at other private locations, such as a friend’s house, while two (3.6%) offenses occurred in public spaces (e.g., a bar or in the street). Five (9.1%) offenses took place in the victim’s home.

**Table 3.** Frequency of In-Person Offense Location.

In-person offense location	ASO (*n* = 34)		CSO (*n* = 21)		Total (*N* = 55)	
	*f*	%	*f*	%	*f*	%
No in-person offense***	1	3.0	17	77.3	18	32.7
Tournament or convention	12	36.4	3	13.6	15	27.3
Offender’s home**	12	36.4	1	4.5	13	23.6
Other private location	9	27.3	1	4.5	10	18.2
Victim’s home	3	9.1	2	9.1	5	9.1
In public	2	6.1	0	0.0	2	3.6

*Note*. ASO = adult sex offenses; CSO = child sex offenses.

** *p* ≤ .01. ***p ≤ .001.

It is worth noting that 18 (32.7%) statements did not outline any in-person offense. This was significantly more common (*p* < .001) in cases involving a child (77.3%) than in adult cases (3.0%). This is unsurprising considering the online offending behavior described above.

### Online Platforms

Online platforms were used at different points throughout the offense process. Table 4 shows how frequently they were used. The platforms mentioned most often across the statements were Discord (34.5%) and Twitch (32.7%). This is unsurprising given both platforms target gamers as their primary demographic. Twitch was used at a similar rate in both adult (33.3%) and child (31.8%) cases; however, Discord was significantly more common (*p* = .003) in cases involving children (59.1%) than those involving adults (18.2%).

**Table 4.** Frequency of Online Platforms Used as Part of Offense Process.

Online platform	ASO (*n* = 34)		CSO (*n* = 21)		Total (*N* = 55)	
	*f*	%	*f*	%	*f*	%
Discord***	6	18.2	13	59.1	19	34.5
Twitch	11	33.3	7	31.8	18	32.7
Twitter	3	9.1	6	27.3	9	16.4
Snapchat**	0	0.0	6	27.3	6	10.9
Skype**	0	0.0	6	27.3	6	10.9
Facebook	2	6.1	3	13.6	5	9.1
Nintendo online*	0	0.0	4	18.2	4	7.3
Instagram	0	0.0	1	4.5	1	1.8
Tinder	1	3.0	0	0.0	1	1.8

*Note*. ASO = adult sex offenses; CSO = child sex offenses.

* *p* ≤ .05. **p ≤ .01. ***p ≤ .001.

While online platforms were not used to meet adult victims, they were used to meet 90.9% of the victims who were children. These initial meetings primarily took place on Discord and Twitch. As can be observed in Table 1, most of the online sexual communication occurred in cases involving children. In these cases, Discord and Skype were the most commonly used platforms for both IM and voice chat, while Snapchat tended to be used for sharing sexual imagery.

## Discussion

This study provides the first empirical overview of the landscape of sexual harm in the video game, streaming, and esports community. While sexual harm has been studied across a range of contexts (e.g., Abbey & Jacques-Tiura, 2011; Wolak & Finkelhor, 2013), it has not yet been considered within this community. It is important to address this gap in knowledge and raise awareness of the risk of sexual harm, as the negative consequences of sexual offense victimization are significant (Dworkin et al., 2017, 2023) and this is a community with many young and vulnerable people. The findings of this study show a range of sexual harm occurring in the video game, streaming, and esports community, including rape, sexual assault, child sexual offenses, grooming, and image-based offenses. Additionally, almost half of the victims discussed how they became aware of other victims of the same offender in the time between being victimized and writing their statement. These findings highlight the significant extent of the problem within this community and the urgent need for protection measures.

A number of findings from the research also demonstrate offense process behaviors that appear to be unique to the video game, live streaming, and esports community, providing an original contribution to the literature base. For example, the identification of tournaments, conventions, and the events and venues linked to them as risky locations has not previously been raised in the literature. Additionally, offenders using their fame status as part of the offense process is not evident in wider research findings. Many of the offenders were live streamers or esports professionals, often with large followings and fans. Victims expressed feeling “lucky” that someone many people (including themselves) look up to was interested in them. This allowed the offender, particularly in cases involving children, to largely bypass some elements of the grooming process, such as establishing trust and rapport (Elliott, 2017; Williams et al., 2013). This has theoretical implications, demonstrating that in some contexts, core elements of the grooming process as it is currently understood do not take place.

One surprising finding was the high number of adult victims involved in streaming. The victims in these cases were often just starting out or had a low number of followers compared to the offender. This suggests well-known (particularly male) streamers target less-known female streamers, offering help and support to gain their trust, before exploiting them. In several cases, the offender even expressed that the victim “owed” them for their help. This study draws attention to platforms such as Discord and Twitch, which are perhaps less known in the online sexual offending literature. Some offenders actively use these platforms’ functions to facilitate the offense process (e.g., offering victims moderator roles in their Twitch channel and/or Discord server to maintain compliance). While further investigation is needed, these findings highlight that platforms need to do more to protect their users.

The frequency of in-person offending behavior was low in cases involving children. This trend can also be found in the broader child sexual offending literature (Villacampa & Gómez, 2017), as children are typically under parental/carer supervision. Instead, offenders tend to use online methods, as they can maintain contact with the victim even when they are at home with their parents/carers. Both online sexual communication and sharing of sexual imagery were common in cases involving children. Additionally, offenders encouraged young people to use “live” communication methods, such as voice chat and video calling, particularly for more serious offending behavior. A possible explanation is that this type of contact leaves less digital evidence for prosecution. When an IM or image is sent, depending on the platform, the content can be viewed and shared. However, voice chat and video calls tend to only leave a time stamp, making it difficult to evidence what took place within the call. Similarly, offenders did not send pornographic media to young people, and only a few sent sexual media of themselves. These tactics are common in the grooming literature and are used to desensitize children to sexual content (DeHart et al., 2017). Given offenders in the sample avoided this approach and showed awareness of leaving minimal digital evidence, this indicates the technological expertise unique to this sample in comparison to other sex offenders. They are more technologically astute due to their time spent in online environments and they bring this knowledge to the offending process.

Alcohol consumption for both the offender and victim was significantly more common in adult cases and is a well-established risk factor in the literature (Seto, 2019). Alcohol can act as a disinhibitor for offenders, weakening cognitive processes and making it difficult to attend to cues that prevent offending behavior (e.g., thinking about future consequences and misperceptions of sexual intentions; Abbey et al., 2004). In this study, they either targeted victims who had already consumed alcohol and/or provided the victim with alcohol. In these cases, the offender and victim often knew each other prior to the offense. Similarly, some victims and offenders were in a romantic relationship with each other at the time of the offense. This supports previous literature that demonstrates many sexual offenses involve an offender and victim who know each other, rather than the misconception that most sexual offenses are committed by strangers (Ullman et al., 2006; Waterhouse et al., 2016).

Additionally, persistent attempts to proposition the victim was the most prevalent offense process characteristic identified in this study and is a common tactic used by grooming offenders (Williams et al., 2013) and domestic sexual assault offenders (Fernet et al., 2021). It creates an environment where the victim feels unable to refuse the offender’s advances. Some offenders also pressured victims to keep their communications secret, a tactic identified in the wider online child sexual grooming literature (Bergen et al., 2014). These findings suggest some similarities between sexual harm in the video game community and broader sexual offense contexts.

The research can also begin to contribute to a theoretical discussion of sexual offending within the video gaming, live streaming, and esports community. While no previous research has explored sexual offending within this context, many of the crimes detailed in the statements have similarities to sexual offenses in other contexts. For example, instances of esports coaches offending against players are similar to coach-athlete sexual offenses observed in traditional sports (Gaedicke et al., 2021). Similarly, esports players and/or live streamers using their position of fame within the community to access victims mimics celebrity sex offending (Lankford et al., 2024). However, research regarding sexual offending in traditional sports and celebrity sexual offending remains in its infancy and lacks an established theory to explain some of the unique factors within these crimes (Gaedicke et al., 2021; Lankford et al., 2024). Despite this lack of theory, Lankford et al. (2024) proposed the Sexual Frustration theory (Lankford, 2021) as a possible explanation for celebrity offending, which may also offer insights into the offending behavior of live streamers and esports players. This theory outlines three types of sexual frustration; (1) unfulfilled desires to have sex, (2) unavailable partners, and (3) unsatisfying sexual activities. This leads to the inability to have sex “when one wants, with whom one wants, how one wants” (Lankford, 2021, p. 4). Lankford (2021) argues that the adoration they receive from fans, coupled with monetary success, can lead to an inflated ego and sense of entitlement to have sex when, with whom, and however they like. These same factors can also be seen with video game live streamers and esports players, albeit to a smaller extent. Both groups sit in a position of fame and have their own community of fans. Their roles typically involve playing video games for extended periods of time (for either content or training), with limited physical contact with others. This could lead to sexual frustration, which may result in sexual offending when in close physical proximity to others from the community (such as tournaments or conventions) or in private online spaces (such as Discord calls or chats).

Another potential theoretical application of the findings could be linking the misogynistic and sexist culture in online video games to established theory of sexual offending. Studies have explored the experiences of female gamers in online gaming spaces, and have found they are met with hostile sexism (Cote, 2017; Fox & Tang, 2014, 2017; Oh & Choi, 2023; Ruvalcaba et al., 2018; Seo et al., 2022). A range of general theories of sexual offending highlights the impact of cultural and social factors as playing an important part in the etiological puzzle (Ward & Beech, 2006). The immersion of video game streamers and esports players in the misogynistic and sexist online gaming culture could offer some explanation for why offenders in this group commit sexual offenses. However, it is acknowledged that cultural factors alone do not provide a sufficient explanation, with genetic and ecological factors also playing a significant role in sexual offending (Ward & Beech, 2006).

### Practical Implications

The findings of the study carry a range of practical implications for the video game, streaming, and esports community. Almost all offenders were engaged in live video game streaming and/or esports. This indicates not only a cultural problem within these two professions that needs to be addressed, but also a lack of safeguards in place to protect viewers, fans, and other competitors. Video game streamers can find themselves in two-way communication with their fans (including children) through live chat functions on platforms. It is difficult to think of other professions that provide an adult with unsupervised contact with children and do not also require the adult to complete significant safeguarding training and/or formal criminal history checks. Additionally, the most common offense process characteristic was persistent attempts to proposition the victim (e.g., for sex, images, and/or sexting). This creates an environment where victims feel unable to reject the offender, as their previous attempts to say “no” were not respected. These issues highlight a gap in understanding and education around healthy relationships and consent within this community that could be addressed at an organizational level. Both streaming and esports organizations offer lucrative contracts to players and provide them with significant platforms to gain fans and viewers. It could be argued, therefore, that these organizations have a duty of care to take steps to educate their players to reduce the potential for sexual harm. This is similar, for example, to the safeguarding training teachers must complete (Walsh et al., 2023) and sexual harassment training many workplaces provide for employees (Campbell et al., 2013).

The high rate of sexual harm occurring while attending tournaments, conventions, and organized after-parties for these events needs to be addressed. Again, this indicates a cultural issue that needs to be acknowledged and tackled by organizers. For example, awareness campaigns promoted through these events can increase vigilance and reporting of sexually aggressive or harmful behavior before offenses are committed. Such campaigns could be similar to those aimed at sexual violence awareness on university campuses (Potter et al., 2009) and in nightlife environments (Quigg et al., 2021).

The study’s findings highlight a gap in sexual education for children and young people in relation to the video game, streaming, and esports community. In the UK, Sex and Relationship Education (SRE) tends to focus on the potential harm strangers pose toward children (i.e., “stranger danger”; Jay et al., 2020). However, live streamers sit in a gray area between strangers and friends. Streaming platforms offer children two-way communication with the person they are watching. This two-way communication instills a sense of familiarity between the child and streamer, such that the child does not view the streamer as a stranger, nor do they perceive any danger. SRE and similar educational programs in other countries should draw attention to this relationship dynamic and highlight that watching a live streamer does not mean they are no longer a stranger.

### Limitations and Future Research

While this study is the first of its kind to explore the landscape of sexual harm in the video game, streaming, and esports community, it has some limitations. The study used a relatively small sample of cases. Although 55 cases is not an insignificant sample, particularly given the sensitivity of the cases, it made more complex statistical analysis difficult due to issues around statistical power. Many of the differences between adult and child cases were approaching significance and would have likely met this threshold with a larger sample. Future research should, therefore, identify a wider sample of cases so the findings of the current study can be validated.

Additionally, the data for this study were extracted from victim statements posted online. A number of these statements have led to investigations where the offender has been found guilty, and others included screenshots and other evidence within the statements to support their accounts. However, some statements had no supporting material, meaning some caution should be taken when reviewing these findings. Like other self-report measures (e.g., interviews), victims were sharing their experiences. As such, they may focus on salient issues to them at the time and may miss out certain details. Therefore, variables coded as “absent” in the study, could potentially have been present, but just not mentioned in the statement. Furthermore, these statements provide a one-sided account of the interactions between two parties. In response to these publicly posted victim statements, many of the live streamers and/or esports professionals offer their own public account, either refuting the claims by the victim or admitting to their actions. Future research could consider analyzing these response statements to understand live streamer and/or esports player responses to public allegations.

Finally, the current research cannot provide insight regarding the prevalence of these types of offenses. Only disclosed accounts of sexual harm within the video game, streaming, and esports community were explored. Future research should conduct large-scale cross-sectional surveys of this community to identify rates of victimization across the population. This will allow insight regarding the extent of sexual harm within this community.

To summarize, this is the first study to explore the landscape of sexual harm and victimization in the video game, live streaming, and esports community. The findings demonstrate offending patterns unique to this community, such as bypassing typical grooming processes, a preference for “live” offense methods that limit digital evidence, and using tournaments and conventions to target victims. This is significant as it highlights the need for bespoke safeguards to protect both adults and children within this community.
